# Lithium-Induced Modulation of Proliferation and Apoptosis in an In Vitro Model of Colorectal Cancer

**DOI:** 10.3390/ijms262211222

**Published:** 2025-11-20

**Authors:** Edgar Yebrán Villegas-Vázquez, Ximena Paola Becerril-Vigueras, Gerardo Leyva-Gómez, Samantha Andrea Porras-Vázquez, Luz Aleida Jiménez-Fernández, Jorge Manuel Almanza-Torres, Lilia Patricia Bustamante-Montes, Miguel Rodríguez-Morales, Virgilio Eduardo Trujillo-Condes, Mariana de la Torre-Núñez, Beatriz Rosario Tinoco-Torres, Nieves Herrera-Mundo, Fátima Elizabeth Murillo-González, Octavio Daniel Reyes-Hernández, Gabriela Figueroa-González

**Affiliations:** 1 Facultad de Estudios Superiores Zaragoza, Universidad Nacional Autónoma de Mexico, Ciudad de Mexico 09230, Mexico; eyebran.villegas@gmail.com; 2 Laboratorio de Farmacogenética, Facultad de Estudios Superiores Zaragoza, UMIEZ, Universidad Nacional Autónoma de Mexico, Ciudad de Mexico 09230, Mexico; ximevigueras1811@gmail.com (X.P.B.-V.); andy.porras13@gmail.com (S.A.P.-V.); aleidafernandezttro@gmail.com (L.A.J.-F.); jorgemanuelalmanzatorres@gmail.com (J.M.A.-T.); 3 Departamento de Farmacia, Facultad de Química, Universidad Nacional Autónoma de Mexico, Ciudad de Mexico 04510, Mexico; leyva@quimica.unam.mx; 4 Coordinación de Investigación, Centro Universitario Siglo XXI, Estado de Mexico 51350, Mexico; patriiab@yahoo.com; 5 Departamento de Genética Humana, Instituto Nacional de Pediatría, Ciudad de Mexico 04530, Mexico; geneticarodriguez@gmail.com; 6 Facultad de Medicina, Universidad Nacional Autónoma de Mexico, Ciudad de Mexico 04510, Mexico; 7 Facultad de Medicina, Universidad Autónoma del Estado de Mexico, Estado de Mexico 50180, Mexico; vetrujilloc@uaemex.mx; 8 Ciencias de la Salud, Universidad Autónoma de Guadalajara, Jalisco 44340, Mexico; mariana.delatorre@edu.uag.mx (M.d.l.T.-N.); beatriz.tinoco@edu.uag.mx (B.R.T.-T.); 9 Departamento de Biología Celular y Fisiología, Instituto de Investigaciones Biomédicas, Universidad Nacional Autónoma de Mexico, Ciudad de Méxio 70228, Mexico; nieves_herrera@iibiomedicas.unam.mx; 10 Diagnóstico Molecular de Leucemias y Terapia Celular (DILETEC), Ciudad de Mexico 07800, Mexico; fatimamurilloglez@gmail.com

**Keywords:** apoptosis, colorectal cancer, drug repurposing, lithium salts, proliferation

## Abstract

Cancer involves uncontrolled cell growth, leading to tumor formation, and remains a major cause of mortality worldwide. Colorectal cancer (CRC) arises from abnormal proliferation of colon glandular epithelial cells. We assessed the cytotoxic and molecular effects of lithium carbonate (Li_2_CO_3_) and lithium chloride (LiCl) in two CRC cell lines (HCT-116 and SW-620) and a non-tumorigenic line (CRL-1790). Viability assays revealed dose-dependent cytotoxicity, with HCT-116 being the most sensitive cell line (IC_50_: 8.14 mM for Li_2_CO_3_). Notably, long-term lithium exposure reduced proliferation, lowering colony-forming efficiency (CFE) and a phenotypic shift from holoclones to meroclones and paraclones, indicating diminished self-renewal capacity. Minimal membrane damage was observed (LDH assay), suggesting non-lytic mechanisms consistent with apoptosis. TUNEL and Annexin-V/IP assays confirmed apoptosis in >40% of cells, without caspase-3 cleavage, suggesting a caspase-independent pathway. PARP-1 cleavage occurred only in SW-620 cells. Western blotting exposed cell-specific modulation of GSK-3β: increased inactive form (p-Ser9) in CRC cells and decreased in CRL-1790 cells, implying differential disruption of Wnt/β-catenin signaling. c-Myc levels remained unchanged, suggesting possible post-translational regulatory effects. Overall, these findings indicate that lithium salts selectively reduce CRC cell viability, impair stem-like characteristics, and induced caspase-independent apoptosis. Therefore, we expand the proof of concept of the potential of lithium-based compounds as low-toxicity adjuvant agents in colorectal cancer therapy.

## 1. Introduction

Cancer is a disease characterized by the uncontrolled growth of the body’s cells, often leading to the formation of malignant tumors in various organs. Colorectal cancer (CRC) arises from the epithelial lining of the colon or rectum and is classified into three main subtypes: hereditary, sporadic, and colitis-associated [1]. The development of this neoplasia is influenced by a combination of genetic and environmental factors, including dietary habits, physical activity, and alterations in the intestinal microbiota, all of which play a significant role in CRC progression and contribute to its status as a growing global health concern. According to GLOBOCAN 2020, approximately 19.3 million new cancer cases and 10 million cancer-related deaths were reported worldwide, with CRC accounting for 1.93 million cases and nearly 0.94 million deaths [2].

Despite advances in chemotherapy, including the use of drugs like doxorubicin, challenges such as drug resistance, toxicity, and tumor relapse persist. Consequently, drug repurposing strategies, such as the use of lithium salts, offer a promising approach by leveraging known pharmacological profiles while uncovering novel anticancer mechanisms. In recent years, lithium salts, traditionally utilized as mood stabilizers in psychiatric disorders, have gained significant interest for their potential anticancer properties. The interest stems from lithium’s ability to modulate key signaling pathways, particularly by inhibiting glycogen synthase kinase-3 beta (GSK-3β), a serine/threonine kinase involved in regulating cell proliferation, differentiation, and apoptosis [3]. Lithium-triggered inhibition of GSK-3β has been demonstrated to slow tumor growth in various cancers, including neuroblastoma, ovarian, and prostate cancer, partly through its effects on β-catenin stability and modulation of the Wnt signaling pathway [3,4,5]. Moreover, preclinical studies have demonstrated that lithium exerts cytotoxic and anti-proliferative effects in colorectal cancer models [6].

The Wnt/β-catenin signaling pathway plays a crucial role in maintaining tissue homeostasis by regulating key processes, including cell fate, proliferation, and apoptosis. However, its aberrant activation, combined with mutations in genes such as APC, CTNNB1 (which encodes β-catenin), or Axin, constitutes a well-established mechanism in the development of CRC [7]. The pathway does not operate in isolation but is closely interconnected with other signaling cascades, such as Notch and KRAS, forming a complex molecular network that promotes tumor progression and contributes to therapeutic resistance [8]. Notably, activation of the Wnt pathway increases the expression of oncogenes such as c-Myc and cyclin D1, thereby supporting uncontrolled cell growth and tumor development [9,10].

Interestingly, Wnt signaling not only promotes proliferation but also influences apoptotic responses. For example, SOX-1-mediated activation of β-catenin increases the expression of proapoptotic targets, including Bcl-2 and caspase-related genes, leading to programmed cell death through both intrinsic and extrinsic pathways [9,11]. The pathways involve the sequential activation of initiator caspases (such as caspase-8 and -9) and effector caspases (such as caspase-3), ultimately resulting in DNA fragmentation, PARP cleavage, and cell death [12,13,14].

Given the urgent need for more effective and less toxic cancer therapies, this study investigates the therapeutic potential of lithium salts (LiCl and Li_2_CO_3_) as anticancer agents in CRC. By examining the cytotoxic, anti-proliferative, and proapoptotic effects of these compounds in CRC cell lines (HCT-116 and SW-620), this study aims to elucidate novel mechanisms of action for these well-characterized compounds. This research provides valuable insights into lithium-induced cell death through a comprehensive analysis of cell viability, membrane integrity, clonogenic potential, apoptotic DNA fragmentation, and the expression of key signaling proteins, including GSK-3β, c-Myc, PARP-1, and pro-caspase-3. The findings could provide new insights into repositioning lithium salts as affordable and accessible therapeutic options, clarify the molecular mechanisms underlying lithium-induced cell death in CRC models, and make them a future therapeutic option in CRC treatment.

## 2. Results

### 2.1. Effect of Lithium Salts on Cell Proliferation

**Figure 1:**
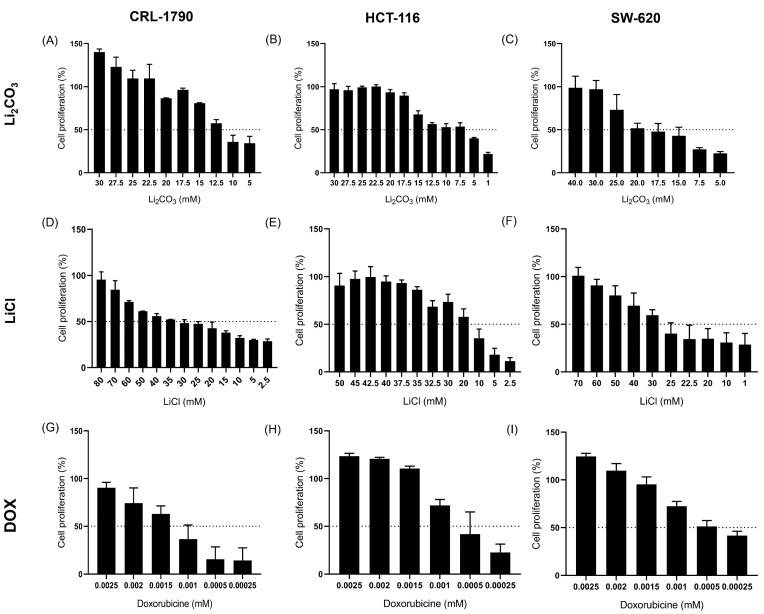
Effect of lithium salts on Cell Proliferation of CCR. Cell lines CRL-1790 (**A**,**D**), HCT-116 (**B**,**E**), and SW-620 (**C**,**F**) were treated with increasing concentrations of Li_2_CO_3_, LiCl, and DOX (CRL-1790 (**G**), HCT-116 (**H**), and SW-620 (**I**) cell lines) for 24 h to assess the anti-proliferative effect of lithium salts. In all three cell lines, lithium sales reduced cell proliferation in a dose-dependent manner, indicating that Li_2_CO_3_ and LiCl exerted anti-proliferative effects. Data are presented as mean ± standard deviation (S.D.) relative to untreated control cells. All experiments were performed in three independent biological replicates with technical triplicates.

CRC cell lines—CRL-1790, HCT-116, and SW-620—were treated with increasing concentrations of Li_2_CO_3_ and LiCl (Figure 1A–F). Results from the SRB assay indicated a progressive, dose-dependent decrease in cell viability, suggesting that lithium salts exert cytotoxic effects on CRC cell lines in a concentration-dependent manner. Additionally, DOX was utilized as a control to assess its impact on CRC cell proliferation (Figure 1G–I). As hypothesized, cell viability decreased in a dose-dependent manner with increasing DOX concentration.

IC_50_ values were calculated applying non-linear regression analysis with a four-parameter logistic model (Table 1). Among the lithium salts tested, HCT-116 cells exhibited the highest sensitivity, with IC_50_ values of 8.14 mM for Li_2_CO_3_ and 16.63 mM for LiCl. In contrast, SW-620 and CRL-1790 cells displayed moderate sensitivity to both lithium compounds.

Treatment with DOX demonstrated significantly greater potency in inhibiting cell proliferation compared to lithium salts across all tested cell lines. The results indicate that while lithium salts induce anti-proliferative effects at millimolar concentrations, DOX is effective at much lower, micromolar to sub-micromolar levels.

### 2.2. Effect of Lithium Salts on Cellular Cytotoxicity

**Figure 2:**
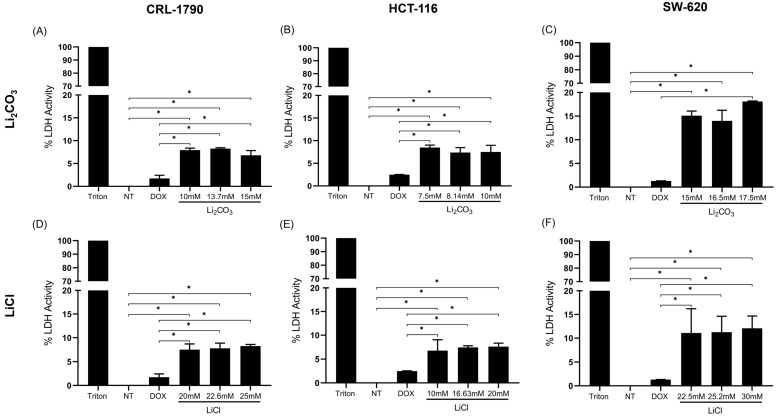
Cytotoxicity effect of lithium salts on CRC cell lines. CRL-1790, HCT-116, and SW-620 cells were treated with Li_2_CO_3_ and LiCl at their IC_50_, high, and low doses. Additional treatments included Triton X-100 (a positive control for necrosis), non-treated cells (NT), and Doxorubicin-treated cells (DOX). Cytotoxicity was assessed using the LDH release assay. Bars represent LDH activity (percentage) in response to each treatment. The dashed line indicates the 20% threshold, which is considered the upper limit for acceptable LDH leakage. Data are presented as mean ± standard error (SE) from three independent experiments, each performed in triplicate. * Statistical significance was *p* < 0.05 according to Tukey’s multiple comparisons test. (Panels (**A**–**C**)) show the effect of Li_2_CO_3_ on CRL-1790 (**A**), HCT-116 (**B**), and SW-620 (**C**) cells, demonstrating a dose-dependent increase in LDH release, indicative of membrane damage. (Panels (**D**–**F**)) illustrate the effect of LiCl on CRL-1790 (**D**), HCT-116 (**E**), and SW-620 (**F**) cells, showing similar trends in cytotoxicity with increasing concentrations.

The LDH assay measures the activity of lactate dehydrogenase (LDH), an enzyme that converts lactate to pyruvate, serving as an indicator of cell membrane damage in cytotoxicity assays. In this study, each assay included non-treated cells (NT), a positive control (cells treated with Triton X-100), and three concentrations of lithium salt (IC_50_, low, and high doses) (Figure 2).

LDH activity was undetectable in the NT group. In contrast, the highest LDH activity was observed in the positive control, indicating elevated cytotoxicity associated with necrotic cell death. In treatments with Li_2_CO_3_ and LiCl, both at IC_50_, reduced LDH activity was detected, remaining below 20% of the total release, which is an accepted threshold indicating preserved membrane integrity and low cellular damage.

For Li_2_CO_3_, the LDH release values were 8.26% ± 0.19%, 7.38% ± 1.07%, and 11.79% ± 4.14% for CRL-1790, HCT-116, and SW-620 cells, respectively. For LiCl, LDH release values were 7.77% ± 1.08%, 7.43% ± 0.33%, and 11.25% ± 3.36% for the same cell lines (Table 2).

Statistical analysis revealed significant differences between lithium salts (Li_2_CO_3_ and LiCl) and non-treated cells (*p* < 0.05), as well as between lithium salts and DOX-treated cells (*p* < 0.05) (Figure 2).

In CRL-1790 cells, statistically significant differences were observed between the untreated group and the doxorubicin treatments, as well as with the IC_50_ concentration of Li_2_CO_3_. A similar pattern was observed in the HCT-116 cell line. In contrast, in the SW-620 cells, significant differences were found only between the control group and the treatment with the IC_50_ concentration of Li_2_CO_3_.

Regarding the LiCl treatment, statistically significant differences were detected between the control and doxorubicin treatments in all three cell lines evaluated (CRL-1790, HCT-116, and SW-620), with additional significant differences also observed when compared to the IC_50_ concentration of LiCl in each case (Figure 2).

The results suggest that neither high nor low concentrations of Li_2_CO_3_ or LiCl cause cytotoxic effects, as measured by LDH release, in CRL-1790, HCT-116, and SW-620 cells.

### 2.3. Long-Term Anti-Proliferative Effects of Lithium Salts

The colony formation assay evaluates a single cell’s ability to grow into a colony, reflecting its long-term proliferative potential. To evaluate this capacity of lithium over time, clonogenic assays were performed in CRL-1790, HCT-116, and SW-620 cells. Treatments were carried out at IC_50_ concentrations of Li_2_CO_3_ and LiCl for 24 h. Cells were grown for 10–14 days, followed by fixation, crystal violet staining, and colony counting.

**Figure 3:**
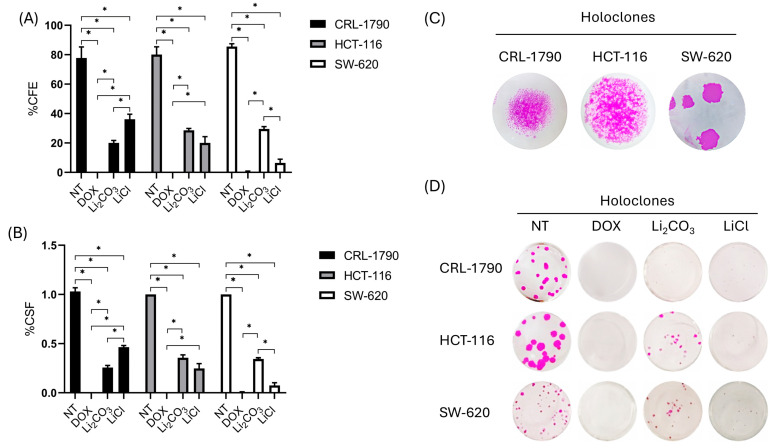
Clonogenic assay results demonstrating decreased survival and increased sensitivity to IC_50_ concentrations of Li_2_CO_3_, LiCl, and doxorubicin in CRL-1790, HCT-116, and SW-620 cell lines. (**A**) Histogram illustrating the percentage of colony-forming efficiency (CFE) in cells treated with lithium salts, doxorubicin, and non-treated controls at 24 h. * Statistical significance was set at *p* < 0.05 according to one-way ANOVA followed by Tukey’s multiple comparisons test. (**B**) Histogram showing the percentage of clonogenic survival fraction (CSF) following 24 h treatment at IC_50_ concentrations of Li_2_CO_3_, LiCl, doxorubicin, and non-treated controls. Bars represent the mean ± standard deviation from three independent biological replicates. * Statistical significance was *p* < 0.05 according to the one-way ANOVA test and Tukey’s multiple comparisons test. (**C**) Representative images exposing the morphological features and density of holoclones, following treatment and colony development arrest. (**D**) Representative photomicrographs of fixed colonies stained with crystal violet, displaying the morphology and density of colorectal cancer cells and colonies after 10 days of culture with or without treatment using lithium salts or doxorubicin.

A significant reduction in colony number and size was observed in both lithium-treated cells compared to untreated and DOX-treated controls. The clonogenic survival fraction (CSF) and colony formation efficiency (CFE) decreased across all cell lines, with statistically significant differences compared to the controls (*p* < 0.05) (Figure 3). CSF and CFE data are summarized in Table 3.

The findings indicate that Li_2_CO_3_ and LiCl significantly impair the long-term proliferative potential of CRC cell lines, although the degree of sensitivity varies depending on the cell line and lithium salt. The differential responses suggest cell line–specific mechanisms of action, possibly linked to variations in stress response pathways, DNA repair capacity, or apoptotic regulation.

Additionally, the self-renewal capacity and pluripotency characteristics of CRC cell lines were evaluated based on the morphology and density of holoclones, meroclones, and paraclones (distinct colony types arising from clonogenic assays). Holoclones are densely packed colonies with smooth, uniform borders and high proliferative potential (Figure 3C,D). Meroclones display intermediate morphology and proliferative capacity, while paraclones form irregular, dispersed colonies and are associated with limited self-renewal capacity.

Colonies formed after 10 days of treatment were classified based on morphological characteristics. In non-treated controls, holoclones were predominant, displaying tightly packed cells with well-defined, smooth borders, indicative of stem-like cells (Figures S1–S3). Following treatment with Li_2_CO_3_ and LiCl at IC_50_ concentrations, a significant reduction in the number of holoclones was observed in all three CRC cell lines (Figure 3). Meroclones and paraclones became more prevalent, indicating a shift toward reduced clonogenic and self-renewal capacity. Statistical analysis revealed significant differences in the proportions of holoclones, meroclones, and paraclones between lithium-treated and control cells across all CRC cell lines (*p* < 0.05) (Figures S1–S3).

### 2.4. Apoptotic Effects of Lithium Salts

#### 2.4.1. TUNEL Assay

**Figure 4:**
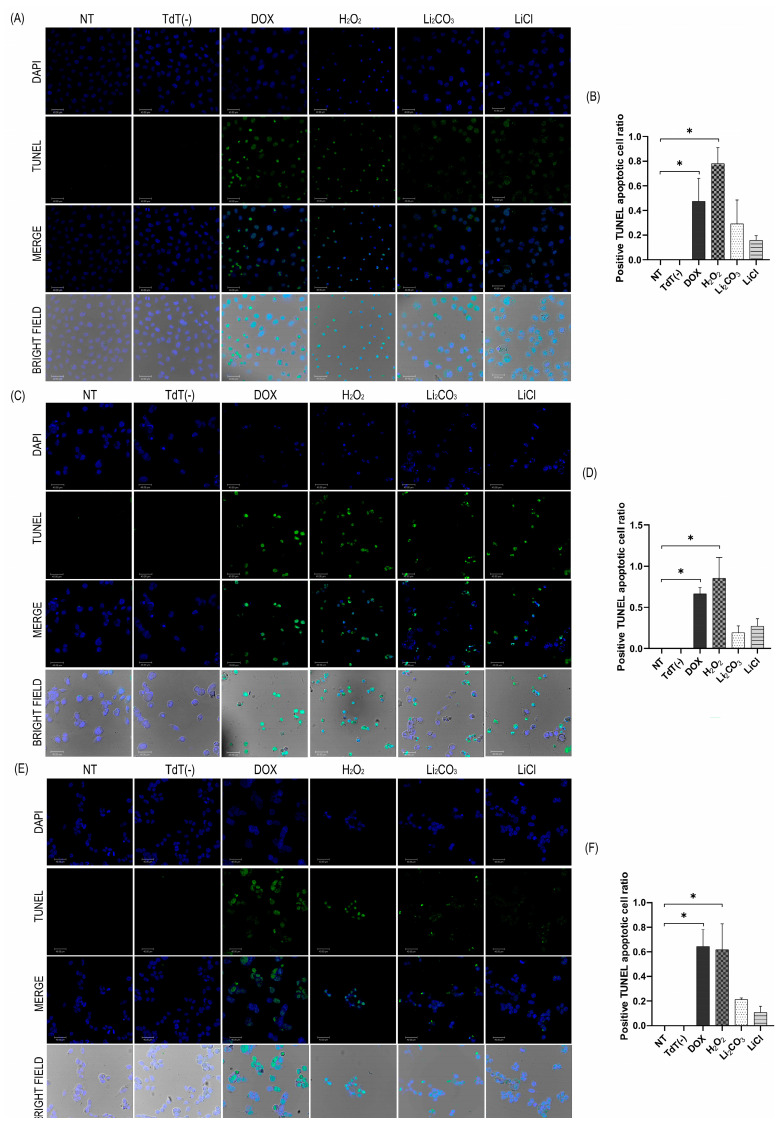
Morphology of DNA fragmentation in cells assessed by fluorescent TUNEL labeling. Panels (**A**,**C**,**E**) (cell lines CRL-1790, HCT-116, and SW-620, respectively), display TUNEL, DAPI, merged, and bright-field images, representing the number of apoptotic cells, determined by the TUNEL assay. A higher number of apoptotic cells was observed in the presence of Li_2_CO_3_ in the SW620 cell line (**E**), while treatment with LiCl induced a greater apoptotic response mainly in the HCT-116 cell line (**C**). Panels (**B**,**D**,**F**) shows the apoptotic cell ratio, in correspondence with their respective composite image (panels **A**,**C**,**E**). The images were obtained using a LEICA confocal microscope with a magnification of 40×. Cells were treated for 24 h under the following conditions: NT, without TdT enzyme (TdT(−)), DOX, H_2_O_2_, Li_2_CO_3_, and LiCl. Each image represents results from three independent experiments. * *p* < 0.05 according to one-way ANOVA followed by Tukey’s multiple comparisons test.

The TUNEL (Terminal deoxynucleotidyl transferase dUTP Nick End Labeling) assay detects DNA fragmentation, a hallmark of the late-stage programmed cell death (apoptosis). TUNEL assays were performed on cell cultures from both control and treated groups to evaluate the extent of apoptosis induced by lithium salts (Figure 4). As expected, positive control samples treated with DOX and H_2_O_2_ exhibited widespread TUNEL positivity across nearly all nuclei, validating the assay’s sensitivity in detecting DNA fragmentation. In contrast, non-treated (NT) control samples and those processed without the TdT enzyme (TdT (−)) produced negligible background fluorescence, indicating a basal level of apoptosis and confirming the specificity of the TdT reaction for 3′-OH DNA ends.

Representative images of cells treated with Li_2_CO_3_ and LiCl revealed a marked increase in TUNEL-positive nuclei, reflecting enhanced DNA fragmentation and pronounced activation of the apoptotic pathways in the CRL-1790, HCT-116, and SW-620 cell lines. Quantitative analysis confirmed a considerable increase in the percentage of apoptotic cells following treatment with both compounds. For Li_2_CO_3_, the proportions of apoptotic cells were 29.1% in CRL-1790, 19.2% in HCT-116, and 21.3% in SW-620. LiCl treatment resulted in apoptotic rates of 15.7%, 27.1%, and 10.5% in the same cell lines, respectively. These values represent marked increases compared with NT control group, supporting the proapoptotic effect of lithium salts in CRC cells.

#### 2.4.2. Annexin V/IP

**Figure 5:**
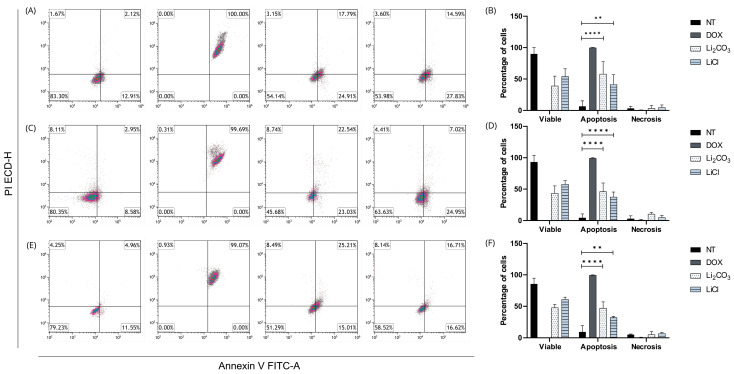
Analysis of apoptosis and necrosis by flow cytometry using Annexin V-FITC/PI dual staining in three human cancer cell lines treated with lithium salts. (**A**,**B**) CRL-1790 cells; (**C**,**D**) HCT-116 cells; (**E**,**F**) SW620 cells. Dot plots illustrate the distribution of viable cells (lower left quadrant: Annexin V^−^/PI^−^), early apoptotic cells (lower right quadrant: Annexin V^+^/PI^−^), late apoptotic cells (upper right quadrant: Annexin V^+^/PI^+^), and necrotic cells (upper left quadrant: Annexin V^−^/PI^+^). Cells were treated with Li_2_CO_3_, LiCl, or doxorubicin (DOX, positive control), or left non-treated (NT). Lithium salt treatments induced a significant increase in apoptotic populations with minimal necrotic response across all cell lines, while DOX induced nearly complete late apoptosis. Data are representative of three independent experiments and the values are expressed as the mean ± SD with ** *p* < 0.0019, and **** *p* < 0.0001.

Flow cytometry analysis using dual staining with Annexin V-FITC and propidium iodide (PI) was conducted to characterize the effects of lithium salts (Li_2_CO_3_ and LiCl) on cell viability and cell death mechanisms, compared with untreated (NT) cells and doxorubicin (DOX) as a positive control (Figure 5). Dot plots display the distribution of viable cells (Annexin V^−^/PI^−^), early apoptotic cells (Annexin V^+^/PI^−^), late apoptotic cells (Annexin V^+^/PI^+^*)*, and necrotic cells (Annexin V^−^/PI^+^).

Across the three cell lines evaluated (CRL-1790, HCT-116, and SW-620; Figure 5A–C), DOX treatment induced near-complete apoptosis, validating the experimental system. In contrast, both lithium salts predominantly promoted apoptosis, with minimal induction of necrosis. Notably, CRL-1790 cells (Figure 5A) exhibited a marked increase in early apoptosis (24.91% with Li_2_CO_3_ and 27.83% with LiCl), while maintaining necrosis levels below 4%, indicating a proapoptotic effect without acute membrane disruption. A similar trend was observed in HCT-116 and SW620 cells (Figure 5B,C), with slight variation in late apoptosis and a minor increase in necrosis observed in Li_2_CO_3_-treated HCT-116 cells.

The results indicate that lithium salts induce programmed cell death in a controlled manner, characterized by low cytotoxicity and preferential activation of apoptotic pathways, highlighting their potential as selective antitumor agents.

#### 2.4.3. Apoptotic Protein Biomarkers

**Figure 6:**
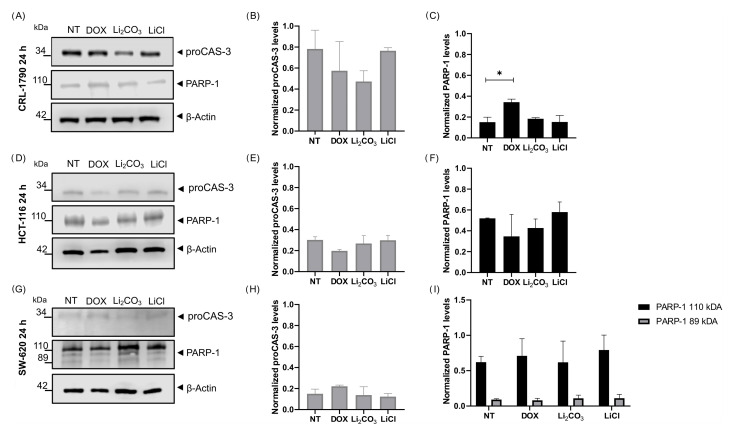
Effect of lithium salts on the expression of apoptotic biomarker proteins after 24 h of treatment. Western Blot analysis of pro-caspase-3 (proCAS-3) and poly(ADP-ribose) polymerase-1 (PARP-1) expression in CRL-1790 (Panels (**A**–**C**)), HCT-116 (Panels (**D**–**F**)), and SW-620 (Panels (**G**–**I**)) cells treated for 24 h with Li_2_CO_3_ or LiCl, compared with NT controls. Full-length proCASP-3 was detected in all cell lines and treatment conditions with no significant changes in expression levels. PARP-1 expression was observed across all groups, showing cell line-specific differences: SW-620 cells exhibited both full-length PARP-1 (110 kDa) and the cleaved fragment (89 kDa), whereas CRL-1790 and HCT116 cells did not display the cleaved form under any treatment condition. β-actin was used as the internal loading control. Data are representative of three independent experiments, and values are expressed as mean ± SD. * *p* < 0.02 according to one-way ANOVA followed by Tukey’s multiple comparisons test.

The expression of apoptotic biomarkers was analyzed to assess the activation of apoptosis following lithium treatment. To further elucidate the molecular mechanisms underlying this response, the expression levels of pro-caspase-3 (proCAS-3) and poly(ADP-ribose) polymerase-1 (PARP-1) were analyzed by Western Blot (Figure 6). In NT control cells, high levels of intact pro-caspase-3 and full-length PARP-1 were detected across all CRC cell lines, suggesting limited basal apoptotic activity under unstimulated conditions.

Treatment with Li_2_CO_3_ and LiCl for 24 h did not induce statistically significant changes in proCAS-3 expression among the cell lines or treatment groups, indicating that caspase-3 cleavage was not prominent under these experimental conditions.

In contrast, PARP-1 expression displayed cell line–specific responses. In SW-620 cells, both full-length (110 kDa) and cleaved fragment (89 kDa) were detected following 24 h of treatment, whereas CRL-1790 and HCT-116 cells did not exhibit the cleaved form under any treatment condition. Statistical analysis of normalized PARP-1 expression revealed a significant difference among treatment groups, as determined by one-way ANOVA followed by Tukey’s multiple comparisons test. β-actin was used as the internal loading control.

### 2.5. Effects of Lithium Salts on Proliferation Protein Biomarkers

**Figure 7:**
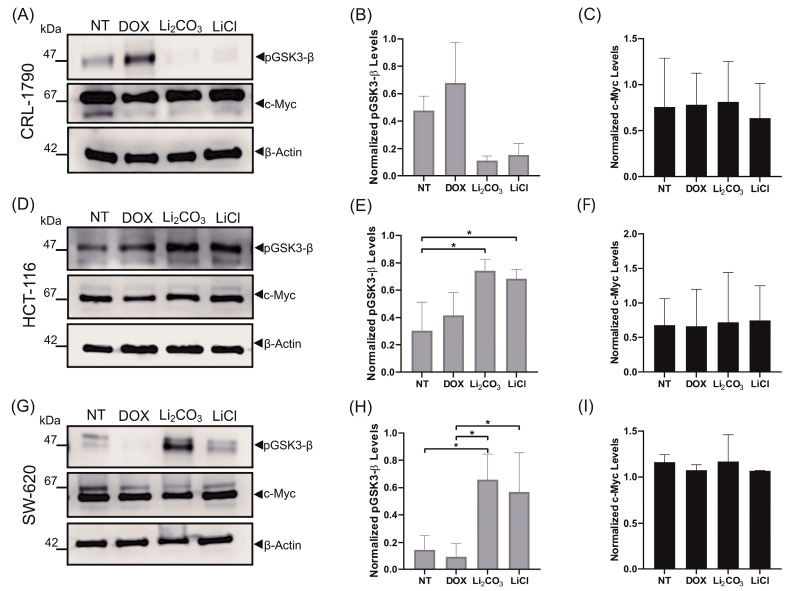
Effect of lithium salts on the expression of proliferation-related biomarker proteins after 24 h of treatment. Western Blot analysis was performed to assess the expression levels of the inactive (Ser9-phosphorylated) form of GSK-3β and c-Myc protein in CRL-1790 (Panels (**A**–**C**)), HCT-116 (Panels (**D**–**F**)), and SW620 (Panels (**G**–**I**)) cells treated for 24 h with lithium chloride (LiCl) or lithium carbonate (Li_2_CO_3_), compared to untreated controls (NT). A decrease in phosphorylated GSK-3β was observed in CRL-1790 cells, whereas its expression increased in HCT-116 and SW620 cells upon treatment with both lithium salts. c-Myc protein expression was consistently detected across all cell lines and treatment conditions. β-actin was used as a loading control. Data represent the mean ± S.D. of three independent experiments. * *p* < 0.02, statistically significant by one-way ANOVA followed by Tukey’s multiple comparisons test.

To assess the impact of lithium salts on signaling pathways associated with cell proliferation, we analyzed the expression levels of the phosphorylated (inactive) form of total glycogen synthase kinase-3 beta at Ser9 (p-GSK-3β) by Western Blot (Figure 7).

In untreated CRL-1790 cells, GSK-3β was predominantly detected in its phosphorylated (inactive) form. In contrast, treatment with Li_2_CO_3_ and LiCl led to a decrease in p-GSK-3β expression in this cell line (Figure 7A–C)

Compared to the controls, densitometric analysis revealed a 4.3-fold decrease in p-GSK-3β expression with Li_2_CO_3_ treatment and a 2.8-fold decrease with LiCl treatment in CRL-1790 (Figure 7 panel B). The reduction suggests reactivation of GSK-3β activity in non-tumorigenic epithelial cells upon lithium exposure.

Conversely, in HCT-116 and SW-620 cells, treatment with both lithium salts resulted in a significant increase in p-GSK-3β levels, indicating GSK-3β inhibition (Figure 7D,G). Specifically, Li_2_CO_3_ induced a 2.3-fold increase in p-GSK-3β in HCT-116 cells (*p* < 0.05) (Figure 7E) and a 4.1-fold increase in SW-620 cells (*p* < 0.05) (Figure 7 panel H). Similarly, LiCl treatment resulted in a 2.1-fold increase in HCT-116 cells (*p* < 0.05) compared to non-treated controls.

To further explore the downstream effects of GSK-3β modulation, we evaluated the expression of the proto-oncogene c-Myc, a known target of GSK-3β that is involved in proliferation and tumorigenesis. Interestingly, no significant changes in c-Myc expression were detected in any of the cell lines following treatment with either lithium salt, compared to their respective controls (Figure 7C,F,I).

## 3. Discussion

**Figure 8:**
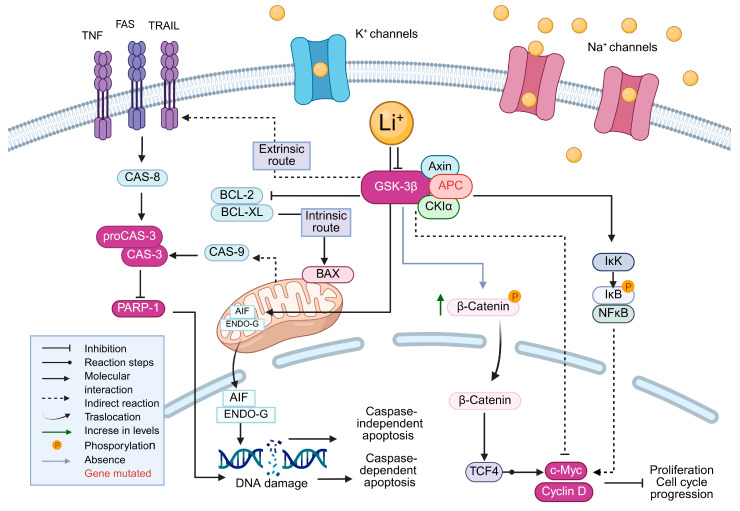
Proposed molecular mechanisms mediated by lithium (Li^+^) in cancer cells. Lithium inhibits GSK-3β, modulating both intrinsic and extrinsic apoptotic pathways, stabilizes β-catenin, and promotes the expression of genes such as c-Myc and Cyclin D. It also influences caspase-dependent and -independent apoptosis, as well as the NF-κB signaling pathway.

The mechanisms by which lithium (Li^+^) interferes with key processes regulating cancer cell survival and apoptosis are illustrated in Figure 8. Lithium acts as a direct inhibitor of glycogen synthase kinase-3 beta (GSK-3β), triggering downstream effects on Wnt/β-catenin, mitochondrial apoptosis, and NF-κB signaling pathways. By inhibiting GSK-3β, the activity of the GSK-3β/CK1α/APC/Axin complex is altered, leading to β-catenin stabilization and nuclear translocation. In the nucleus, β-catenin forms a complex with TCF4, which promotes the transcription of proliferation-associated genes, such as c-Myc and cyclin D1. However, the proliferative signals can be counteracted by lithium-induced apoptosis through mitochondrial pathways, particularly in tumor cells with disrupted Wnt signaling regulation, suggesting a dual role for lithium in modulating cell viability depending on the tumor context [15,16,17].

Our findings demonstrate that Li_2_CO_3_ and LiCl exert dose-dependent cytotoxic effects on colon cell lines (CRL-1790, HCT-116, and SW-620), as shown by SRB assays. Both lithium salts reduced cell viability at higher concentrations, with IC_50_ values in the millimolar range, consistent with prior studies on lithium’s anticancer effects [18,19]. Among the lines tested, HCT-116 exhibited the greatest sensitivity to Li_2_CO_3_ (IC_50_ = 8.14 mM), possibly due to mutations in p53 and β-catenin, which may increase susceptibility to disruption of the GSK-3β/Wnt axis [20,21]. In contrast, SW-620 presented greater resistance, likely reflecting its more aggressive phenotype and adaptation to proliferative signaling [22].

The variable responses to lithium salts among HCT-116, SW-620, and CRL-1790 cells likely reflect their distinct genetic and phenotypic profiles. HCT-116 harbors *KRAS* and *CTNNB1* mutations that activate RAS/MAPK and Wnt/β-catenin pathways linked to SK-3β regulation, making it more susceptible to lithium-induced mitochondrial stress and apoptosis [23,24,25]. In contrast, SW-620, derived from a metastatic site, displays survival adaptations—anoikis resistance, proteomic remodeling, and deregulated Bcl-2/Bim modulation—contributing to lithium resistance and reliance on autophagy [26]. Meanwhile, the non-tumorigenic CRL-1790 line, with stable oxidative metabolism and absence of CRC oncogenic mutations, shows predominantly cytostatic rather than pro-apoptotic response, displaying a moderate sensitivity, in line with previous findings showing lithium’s effects are more pronounced in tumorigenic than in non-tumorigenic cells [27,28].

In comparison, DOX, a well-known chemotherapeutic agent, demonstrated significantly greater potency by inhibiting cell viability at micromolar to sub-micromolar concentrations across all cell lines. This aligns with DOX’s mechanism of action, which involves DNA intercalation and topoisomerase II inhibition, leading to apoptosis [29,30]. However, DOX’s clinical use is limited by cardiotoxicity and chemoresistance, underscoring the need for adjunctive or alternative therapies [31].

Although the requirement for millimolar concentrations of lithium to achieve cytotoxicity poses translational challenges, its low systemic toxicity and ability to modulate key survival pathways make it an appealing candidate for combination therapies. Indeed, lithium has been shown to enhance the efficacy of conventional chemotherapeutics in various cancer models [32,33], and its role in targeting cancer stem cell populations via Wnt signaling remains an area of active investigation [34].

Our results demonstrate that lithium salts, Li_2_CO_3_ and LiCl, affect CRC cell lines by impairing long-term proliferative potential and reducing the self-renewal capacity of clonogenic populations, without inducing significant necrotic membrane damage, as evidenced by LDH release assays. These findings suggest that lithium compounds exert non-lytic cytostatic or apoptotic effects, consistent with prior studies indicating that lithium can modulate survival and proliferation pathways in cancer cells [20,35].

LDH release was minimal across all lithium-treated groups, remaining well below the 20% threshold typically associated with significant cytotoxicity [36]. The results indicate that plasma membrane integrity is maintained, even at IC_50_ concentrations, supporting the idea that cell death is not primarily necrotic. Lithium’s ability to decrease cancer cell viability without causing substantial membrane disruption aligns with its known mechanisms of action, which include modulation of apoptosis, autophagy, and cell cycle checkpoints [37,38].

Lithium treatment impaired long-term proliferation, significantly reduced CFE and CSF, and induced a shift from holoclones to meroclones and paraclones, indicative of suppression of cancer stem cell (CSC)-like properties [39]. These effects occurred without substantial LDH release, supporting the notion that apoptosis rather than necrosis was involved [36]. Cancer stem cells are strongly linked to clonogenic activity, a marker of undifferentiated tumor-initiating populations; the clonogenic assay visualizes the ability of a single cell to form a colony [40,41]. Our findings therefore suggest that lithium may target CSC subpopulations with tumor-initiating potential. This interference provides an advantage over conventional chemotherapy, which is often limitated by drug efflux transporter overexpression and enhanced DNA repair capacity [42].

These results suggest that lithium salts, Li_2_CO_3_ and LiCl, induce apoptosis in CRC cell lines through DNA fragmentation and alterations in cell signaling, while exhibiting distinct effects on proapoptotic markers and signaling kinases, depending on the cell line context. The results support previous studies highlighting the proapoptotic and anti-proliferative effects of lithium in various cancer models [6,43].

TUNEL assays confirmed lithium-induced DNA fragmentation across all CRC lines, with Li_2_CO_3_ exhibiting greater efficacy than LiCl. Interestingly, cleaved caspase-3 was not detected, suggesting caspase-independent apoptosis possibly mediated by the mitochondrial release of apoptosis-inducing factor (AIF) and endonuclease G (ENDOG) [44,45]. Cleavage of PARP-1 was observed only in SW-620, indicating differential apoptotic responses across cell types. The findings align with previous observations that lithium induces mitochondrial stress and non-canonical apoptosis in cancer models [38].

Western Blot analysis revealed context-specific modulation of p-GSK-3β: decreased levels in CRL-1790 and increased levels in HCT-116 and SW-620. The findings imply that lithium may exert an anti-proliferative effect in non-malignant cells, while promoting apoptosis in tumor cells, likely through stabilization of β-catenin and alterations in metabolic homeostasis [20,46]. In contrast, the HCT-116 and SW-620 cell lines showed a notable increase in p-GSK-3β levels, indicating effective inhibition of this kinase, which promotes β-catenin stabilization and may lead to cell cycle arrest [47].

Although GSK-3β is involved in the proteasomal degradation of c-Myc, our results did not show significant changes in the expression of this oncoprotein after lithium treatment. The phenomenon could be attributed to compensatory mechanisms or transcriptional dampening, particularly in cancer cells with deregulated MYC loci [48]. Another possibility is that post-translational modifications of c-Myc—such as phosphorylation or acetylation, which were not addressed in this study—are modulating its function without affecting its total expression levels [49,50].

Furthermore, lithium inhibited NF-κB signaling by preventing IκB phosphorylation, which reduced the transcription of survival genes. There are few studies addressing the molecular mechanisms underlying the effects of lithium in cancer. Among them, only a limited number focus on the impact of lithium on the production of reactive oxygen species (ROS) that exceed the cellular antioxidant capacity. Therefore, based on this evidence, it appears that increased ROS generation and oxidative stress play a central role in the cytotoxic mechanism of lithium. In the study by Li et.al. (2014) [6], an increase in ROS generation was demonstrated at different concentrations of LiCl in SW-480 colon cancer cells (a different cell line from the one used in our study) through the ROS/GSK-3β/NF-κB pathway, thereby suppressing cell proliferation. Another study also reported the induction of cell death through oxidative stress caused by increased ROS levels following LiCl treatment in lung cancer cells [37]. These findings further support the notion that lithium’s cytotoxicity is mediated, at least in part, by redox imbalance and the modulation of GSK-3β/NF-κB signaling.

The effects, illustrated in Figure 8, highlight the lithium-induced reprogramming of oncogenic pathways, as well as the induction of apoptosis in CRC cells.

In summary, lithium salts impair the viability, pluripotency, and survival of CRC cells by inhibiting GSK-3β, inducing mitochondrial stress, and disrupting apoptotic pathways.

Despite higher IC_50_ values compared to conventional chemotherapeutics, their low toxicity and ability to modulate multiple oncogenic pathways make them promising adjuvant candidates. The results align with prior reports and reinforce lithium’s therapeutic potential in CRC treatment [33].

In addition to the in vitro findings presented here, several in vivo and clinical studies support lithium’s antitumor potential by modulating apoptosis, proliferation, and tumor progression. In CRC xenografts, Li_2_CO_3_ reduced metastasis and lymphangiogenesis through TGFBIp/Smad3/GSK-3β inhibition [51]. LiCl has been shown to induce context-dependent autophagy and enhance 5-FU sensitivity [52], promote TNF-α/FasL-dependent apoptosis [53], and inhibit growth via CHOP/NOXA/Mcl-1 activation [54]. Clinically, lithium demonstrated disease stabilization but limited responses in neuroendocrine tumors [55].

Certain limitations of this study must be acknowledged. The experiments were conducted exclusively in vitro, which may not fully emulate the complex tumor microenvironment and systemic interactions occurring in vivo. Moreover, the evaluation focused on short-term exposure and a limited panel of molecular markers. Nevertheless, the emerging evidence supporting the anticancer potential of lithium salts highlights their promise as low toxicity adjuvant agents in experimental oncology. Future work should include in vivo validation to determine optimal dosing and chronic exposure effects, as well as combinatorial approaches with conventional chemotherapeutics or target inhibitors (e.g., Wnt/β-catenin, PI3K/AKT, immune checkpoint pathways) to evaluate synergistic interactions. In addition, the exploration of oxidative stress regulation, mitochondrial-mediated apoptosis, metabolic reprogramming and cancer stem-cell dynamics, alongside advanced molecular omics-based analyses, may help identify lithium-responsive pathways and predictive biomarkers. Ultimately, orthotopic and patient-derived xenograft models will be essential to support the translational potential of lithium salts as adjuvant agents in colorectal cancer therapy.

## 4. Materials and Methods

### 4.1. Cell Culture

Human colon cell lines—including CRL-1790 (Adherent resemble epithelial cell line isolated from colon tissue of a 21 weeks of gestation healthy donor), HCT-116, and SW-620 (colorectal cancer)—were obtained from the American Type Culture Collection (ATCC, Manassas, VA, USA). Cells were cultured in RPMI-1640 medium (Laboratorios MICROLAB S.A. de C.V., M-221P, Mexico City, Mexico), supplemented with 10% neonatal calf serum (NCS) and an antibiotic/antimycotic solution at a final concentration of 10 mL/L. Cultures were maintained under sterile conditions in a humidified incubator (NUAIRE, NU-8600, Plymouth, MN, USA) at 37 °C with 5% CO_2_ and 90% humidity, and were grown until they reached approximately 80% confluence.

### 4.2. Reagents

Lithium chloride (LiCl; 423.9 mg (Sigma-Aldrich, Milwaukee, WI, USA)) and lithium carbonate (Li_2_CO_3_; 442.4 mg (Sigma-Aldrich, St. Louis, MO, USA)) were each dissolved in 50 mL of fresh sterile culture medium and filtered through a 0.2 µm membrane to prepare stock solutions (200 mM for LiCl and 120 mM for Li_2_CO_3_). The sterile stock solutions were then added to the culture medium at the desired final concentrations for each experiment.

Doxorubicin (DOX; 136 µL (Zuclodox, Zurich Pharma, Mexico City, Mexico)) was dissolved in 50 mL of fresh, sterile culture medium and filtered through a 0.2 µm membrane to achieve a final stock concentration of 10 µM. The stock solution was utilized in cytotoxicity assays to determine the IC_50_ value.

Hydrogen peroxide (H_2_O_2_; 1.27 µL (Alcomex, Mexico City, Mexico)) was added to 1 mL of fresh, sterile medium and filtered through a 0.2 µm membrane to achieve a final concentration of 125 µM, which was then directly added to the cell cultures.

### 4.3. Cell Viability Assay and IC_50_ Determination

CRL-1790, HCT-116, and SW-620 cells were seeded in 96-well plates at a density of 7500 cells/well for CRL-1790 and SW-620, and 10,000 cells/well for HCT-116. Cells were incubated for 24 h at 37 °C in a humidified atmosphere containing 5% CO_2_. Subsequently, each cell line was treated with increasing concentrations of LiCl (1–80 mM), Li_2_CO_3_ (1–80 mM), or doxorubicin (DOX; 0.2 × 10^−3^–2.5 × 10^−3^ mM) for an additional 24 h. Untreated CRL-1790, HCT-116, and SW-620 cells were used as controls.

After 24 h of exposure to the lithium salts or DOX, the culture medium was removed, and the cells were fixed with 100 µL of 10% trichloroacetic acid (TCA) for 1 h at 4 °C. The fixed cells were then washed 3–5 times with distilled water and air-dried. Subsequently, 100 µL of 0.4% sulforhodamine B (SRB) staining solution (MP Biomedicals, San Diego, CA, USA) was added per well and incubated for 30 min at room temperature. Excess dye was removed by washing 3–5 times with 1% acetic acid, followed by air drying. The bound dye was then solubilized with 100 µL of 10 mM Tris base (pH 10.5; Promega, Madison, WI, USA) under gentle agitation for 10 min. The Optical density (OD) was measured at 564 nm using a BioTek EPOCH microplate reader (Agilent Technologies, Santa Clara, CA, USA).

Cell viability was quantified based on the amount of SRB dye bound to cellular proteins. Inhibition of cell proliferation was calculated relative to untreated controls using the following equation.

(1) $$Inhibition\;(\%)=\left(\frac{{OD}_{sample}-{OD}_{blank\;sample}}{{OD}_{negative\;control}-{OD}_{blank\;negative\;control\;}}\right)\times 100$$

IC_50_ values were determined through non-linear regression using a four-parameter logistic model. All experiments were performed in three independent biological replicates, each consisting of technical triplicates. Data analysis was conducted using GraphPad Prism software version 8.0.

### 4.4. Lactate Dehydrogenase (LDH) Release Assay

7500 cells/well of CRL-1790 and SW620 cells, and 10,000 cells/well of HCT-116, were seeded in 96-well plates and then incubated at 37 °C in a humidified atmosphere with 5% CO_2_ for 24 h. Cells were treated with their respective IC_50_ concentrations of LiCl and Li_2_CO_3_. Lithium concentrations between 10% and 15% higher and lower than the IC_50_ were used for comparative analysis. After 23 h of treatment, 10 µL of 9% Triton X-100 was added to the positive control wells (necrosis control), and the plates were incubated for an additional hour. Subsequently, the plates were centrifuged at 1600× *g* for 5 min at 4 °C. The supernatants were collected, transferred to new 96-well plates, protected from light, and stored at 4 °C until analysis.

A fresh substrate mix was prepared containing 1.8 mg/mL iodonitrotetrazolium chloride (INT), 1.2 mg/mL 1-methoxy-5-methylphenazinium methyl sulfate (PMS), 2.4 mg/mL β-nicotinamide adenine dinucleotide sodium salt (NAD^+^), 2.3 mg of lithium L-lactate dissolved in 0.2 mL of distilled water, and 2.3 mL of Tris base buffer. The mixture was protected from light and stored at 4 °C until use.

For the LDH assay, 50 µL of the substrate mix was added to each well and incubated at room temperature in the dark for 10–30 min. The reaction was stopped by adding 100 µL of 1 M acetic acid. Absorbance was measured at 490 nm.

Three independent biological replicates, each with technical triplicates, were performed for every experimental condition. Results were expressed as a percentage of LDH activity relative to the positive control, using the following equation:

(2) $$LDH\;activity\;(\%)=\left(\frac{{OD}_{sample}-{OD}_{blank\;sample}}{{OD}_{positive\;control}-{OD}_{blank\;positive\;control}}\right)\times \;100$$

### 4.5. Clonogenic Assay

Human cell lines CRL-1790, HCT-116, and SW-620 were seeded at low density (10–30 cells/well) in 96-well plates that contained complete RPMI-1640 medium supplemented with 10% NCS. The plates were incubated at 37 °C in a humidified atmosphere with 5% CO_2_.

After 24 h, allowing for cell attachment, the cells were treated with Li_2_CO_3_ and LiCl at their respective IC_50_ concentrations. A non-treated cell (medium only) and DOX at its IC_50_ were included. Treatments were repeated on days 4 and 8 to simulate chronic exposure.

Colony formation was monitored on days 4, 6, 8 and 10. On day 10, cells were fixed with TCA 10% (*w*/*v*) for 1 h at 4 °C, later were stained with SRB 0.057% (*w*/*v*) dissolved in acetic acid 1% for 30 min at room temperature.

Wells were gently rinsed with distilled water to remove excess dye and then air-dried. Cell colonies (≥50) were manually counted using an inverted microscope (VWR International, Radnor, PA, USA), following the criteria established to define clonogenic survival as the capacity of a single cell to form a colony. All images were acquired under identical conditions using a Carson Microbrite Plus microscope (Keyence, Osaka, Japan) with a 120× objective.

The number of colonies per well was recorded and normalized to the untreated control. All experimental conditions were conducted in at least three independent biological replicates. Data were analyzed using GraphPad Prism 8.0 software, and colony-forming efficiency (CFE) and clonogenic survival fractions (CSF) were calculated as follows:

(3) $$CFE\;(\%)=\left(\frac{Number\;of\;colonies\;formed}{Number\;of\;cells\;plated}\right)\times \;100$$

(4) $$CSF\;(\%)=\left(\frac{Colonies\;formed\;after\;treatment}{Cells\;plated\times CFE\;of\;control}\right)\times 100$$

### 4.6. TUNEL Assay for Detection of Apoptotic DNA Fragmentation

CRL-1790, HCT-116, and SW-620 colon cell lines (5 × 10^4^ cells/well) were seeded into 24-well plates containing sterile glass coverslips and incubated for 24 h at 37 °C in a humidified atmosphere with 5% CO_2_. After incubation, the cells were treated with LiCl and Li_2_CO_3_ at IC_50_, as previously determined. Additionally, all cell lines were exposed to 125 µM H_2_O_2_ and DOX at their respective IC_50_ values as positive controls. Untreated cells (NT) were included as negative controls. Treatments were conducted for 24 h.

Following treatment, cells were fixed in 4% paraformaldehyde in 0.1 M PBS (pH 7.4) at room temperature for 30 min, then washed with PBS. Permeabilization was conducted using 0.1% Triton X-100 in 0.1% sodium citrate for 2 min at 4 °C, followed by two washes with PBS. Coverslips were then transferred to microscope slides, and 15 µL of TUNEL reaction mixture (prepared according to the manufacturer’s instructions, In Situ Cell Death Detection Kit, Fluorescein 11684795910 (Roche, Mannheim, Germany) was applied to each sample. Samples were covered with coverslips and incubated in a humidified dark chamber at 37 °C for 1 h.

After incubation, samples were washed twice with PBS and counterstained with 30 µL of DAPI (1:500 dilution in PBS; (Calbiochem Gibbstown, NJ, USA)) for 15 min at room temperature in the dark. A final PBS wash was performed prior to imaging. Fluorescence analysis was performed on a TC SP8 confocal microscope DMI8 (Leica Microsystems, Wetzlar, Germany), with excitation wavelengths of 450–500 nm and detection in the range of 490–600 nm (green fluorescence). All images were acquired under identical conditions using a 40× objective, with a 40 µm scale bar included in the lower-left corner for consistency. Image analysis and quantification of DNA fragmentation were performed using LAS X Office software version 1.4.5.27713 (Leica Microsystems, Wetzlar, Germany), which identified apoptotic nuclei based on TUNEL positivity and nuclear morphology.

### 4.7. Annexin V-FITC/PI Flow Cytometry Assay for Apoptosis Detection

CRL-1790, HCT-116, and SW-620 cells (5 × 10^5^ cells/well) were seeded in a 60 mm × 15 mm culture dishes, and after 24 h of growth, the cells were stimulated with the IC_50_ of lithium salts: CRL-1790 cells were treated with 22.6 mM LiCl and 13.7 mM Li_2_CO_3_; HCT-116 cells with 16.63 mM LiCl and 8.14 mM Li_2_CO_3_; and SW-620 cells with 25.21 mM LiCl and 16.5 mM Li_2_CO_3_ for 24 h, respectively. Additional conditions included stimulation with IC_50_ DOX, 125 µM H_2_O_2_, and a non-treated control (NT).

After the stimulation period, the FITC Annexin V Apoptosis Detection Kit II 556547 (BD Biosciences, San Diego, CA, USA) was used to assess apoptotic and necrotic cell death according to the manufacturer’s instructions. Briefly, non-adherent cells were collected from the supernatant, while adherent cells were washed twice with PBS, detached using Versene-EDTA, and pooled with the corresponding suspended cells. Each sample was stained with 1 µL of FITC-conjugated Annexin V and 0.5 µL of propidium iodide (PI) in 100 µL of binding buffer. The samples were then incubated for 10 min at room temperature in the dark and subsequently diluted with 400 µL of PBS.

Samples were acquired using a CytoFLEX S cytometer (Beckman Coulter, Brea, CA, USA), and data were analyzed with Kaluza C software version 1.1 (Beckman Coulter, Brea, CA, USA).

### 4.8. Western Blot Analysis

CRL-1790, HCT-116, and SW-620 cells were cultured and treated for 24 h under different experimental conditions, including lithium salts (LiCl and Li_2_CO_3_) at their respective IC_50_ concentrations, DOX at its IC_50_, and non-treated cells. Following treatment, cells were harvested and lysed in RIPA buffer sc-24948 (Santa Cruz Biotechnology, Dallas, TX, USA) supplemented with a Complete protease inhibitor cocktail 11697498008 (Roche, Basel, Switzerland). Cell pellets were collected in 1.5 mL microcentrifuge tubes and subjected to mechanical lysis by vortexing for 30 s, followed by incubation on ice for 5 min. The cycle was repeated over a period of 30 min. Lysates were centrifuged at 13,000× *g* for 25 min at 4 °C (or 2500 rpm for 20 min as indicated), and the supernatants were transferred to new tubes.

Protein concentrations were quantified using the Bradford protein assay, with spectrophotometric absorbance measured at 595 nm in a Multiskan FC 357 spectrophotometer (Thermo Scientific, Waltham, MA, USA). Equal amounts of protein (50 µg per sample) were mixed with 2× or 5× Laemmli buffer (10% SDS, 50% glycerol, 0.02% bromophenol blue, 0.3125 M Tris-HCl, pH 6.8) supplemented with β-mercaptoethanol and denatured by boiling for 10 min at 100 °C.

SDS-PAGE resolved samples on polyacrylamide gels of varying concentrations, depending on target protein: 6% (PARP-1), 15% (Caspase-3, pGSK3-β, and c-Myc). Gels were transferred to 0.2 µm PVDF membranes 88518 (Thermo Scientific, Waltham, MA, USA) using a Mini Trans-Blot^®^ Cell system 1703810 (Bio-Rad, Hercules, CA, USA) at a constant current of 400 mA for 2 h.

Membranes were blocked in 5% non-fat dry milk in TBS-T (Tris-buffered saline with 0.1% Tween-20) for 2 h at room temperature. Primary antibody incubations were performed overnight at 4 °C under constant agitation in TBS-T with 1% milk, using the following dilutions: PARP-1 1:3000 (Rabbit pAb, A0942 (ABclonal, Woburn, MA, USA)), Caspase-3 1:1000 (Mouse mAb, sc-7272 (Santa Cruz Biotechnology, Dallas, TX, USA)), pGSK3-β 1:1000 (Mouse mAb, sc-11757 (Santa Cruz Biotechnology, Dallas, TX, USA)) and c-Myc 1:1000 (Mouse mAb, sc-40 (Santa Cruz Biotechnology, Dallas, TX, USA)) and β-actin 1:10,000 (Mouse mAb, AC004 (ABclonal, Woburn, MA, USA)) as a loading control.

After three to five washes with TBS-T, membranes were incubated for 1 h at room temperature with HRP-conjugated secondary antibodies: anti-rabbit 1:10,000 (IgG, 7074s (Cell Signaling Technology, Danvers, MA, USA) or anti-mouse 1:10,000 (IgG, 7076s, Cell Signaling Technology, Danvers, MA, USA), followed by additional washes with TBS-T.

Detection was carried out using Clarity Max Western ECL substrate, 1705062 (Bio-Rad, Hercules, CA, USA), and signal visualization was performed with the C-DiGit Blot Scanner (LI-COR, Lincoln, NE, USA). All experiments were performed in biological triplicate. Semi-quantitative densitometric analysis was conducted using ImageJ software (version 7). Data were normalized by dividing the band intensity of each target protein by that of the corresponding β-actin band.

### 4.9. Statistical Analysis

All experiments were conducted in triplicate using at least three independent biological replicates. Data were expressed as the mean *±* standard deviation (SD) or standard error (SE), as appropiate. Statistical analyses were performed using GraphPad Prism software (version 8.0, San Diego, CA, USA).

For comparison between multiple goupr, one-way analysis of variance (ANOVA) was applied, followed by Tukey’s multiple comparisons post-hod test to determine statistical significance. Non-linear regression analysis with a four-parameter logistic model was used to calculare IC_50_ values for Lithium Carbonate (Li_2_CO_3_), Lithium Chloride (LiCl), and doxorubicin (DOX) from cell viability data obtained through SRB assays.

Densitometric analysis of Western Blot bands were normalized to β-actin expression prior to statistical evaluation. Differences in all experiments were considered statistically significant at *p* < 0.05.

## 5. Conclusions

Lithium salts exert dose-dependent cytotoxic effects in CRC cell lines by inhibiting GSK-3β, promoting apoptosis, and impairing clonogenic potential and stem-like cell properties. Despite higher concentrations than conventional chemotherapeutics, their low systemic toxicity and selective modulation of oncogenic signaling pathways highlight their potential as safe adjuvant agents. Importantly, our findings provide new mechanistic insights into lithium’s actions, revealing it ability to reprogram survival and differentiation pathways beyond canonical apoptosis. The observed disruption of cancer stem cell-like subpopulations underscore lithium’s capacity to target tumor heterogeneity and resistance, both features critical to improving therapeutic outcomes. Together, these results support the therapeutic relevance and valuable contribution to elucidating the molecular basis of lithium’s antitumor effects and support its further exploration as a repositioned compound in oncology. Further preclinical and translational studies should expand on these mechanisms to optimize lithium-based strategies for safer and more effective CRC treatment.

## Figures and Tables

**Table 1:** Half inhibitory concentration of lithium salts on CRC cell lines.

Cell Line	IC_50_ Values (mM)		
	Li_2_CO_3_	LiCl	Doxorubucin
CRL-1790	13.70 ± 0.62 mM	22.60 ± 5.60 mM	11.76 × 10^−4^ ± 0.83 mM
HCT-116	8.14 ± 0.81 mM	16.63 ± 0.06 mM	7.98 × 10^−4^ ± 0.77 mM
SW-620	16.5 ± 0.31 mM	25.21 ± 1.90 mM	7.13 × 10^−4^ ± 0.76 mM

**Table 2:** LDH release values of lithium salts on CRC cell lines.

Cell Line	LDH Values (%)		
	Li_2_CO_3_	LiCl	Doxorubucin
CRL-1790	8.26 ± 0.19%	7.77 ± 1.08%	1.72 ± 0.69%
HCT-116	7.38 ± 1.07%	7.43 ± 0.33%	2.49 ± 0.04%
SW-620	11.7 ± 4.14%	11.2 ± 3.36%	1.29 ± 0.02%

**Table 3:** Colony-forming efficiency of CRC cell lines treated with lithium salts.

Cell Lines	Colony-Forming Efficiency (CFE)				Clonogenic Survival Fractions (CSF)			
	Li_2_CO_3_	LICl	DOX	Negative Control	Li_2_CO_3_	LICl	DOX	Negative Control
CRL-1790	20.0 ± 1.6%	36.1 ± 3.4%	0	77.7 ± 7.5%	0.25 ± 0.02%	0.46 ± 0.01%	0	1.03 ± 0.03%
SW-620	29.5 ± 1.5%	6.4 ± 2.5%	0.5 ± 0.4%	85.5 ± 1.9%	0.34 ± 0.01%	0.07 ± 0.02%	0.005 ± 0.004%	1.00 ± 0.00%
HCT-116	28.6 ± 1.2%	20.0 ± 4.3%	0	80.1 ± 5.2%	0.35 ± 0.01%	0.24 ± 0.04%	0	1.00 ± 1.20%

## Data Availability

The original contributions presented in this study are included in the article/Supplementary Material. Further inquiries can be directed to the corresponding author(s).

## Supplementary Materials

The following supporting information can be downloaded at: https://www.mdpi.com/article/10.3390/ijms262211222/s1.

## Abbreviations

The following abbreviations are used in this manuscript:

CRC	Colorectal Cancer
Li_2_CO_3_	Lithium Carbonate
LiCl	Lithium Chloride
CFE	colony-forming efficiency
GSK-3β	glycogen synthase kinase-3 beta
LDH	lactate dehydrogenase
CSF	Clonogenic survival fraction
TUNEL	Terminal deoxynucleotidyl transferase dUTP Nick End Labeling
ENDOG	endonuclease G
AIF	apoptosis-inducing factor
