# Supplementary material for: Lithium-Induced Modulation of Proliferation and Apoptosis in an In Vitro Model of Colorectal Cancer
Source: Int J Mol Sci. 2025 Nov 20;26(22):11222. doi: 10.3390/ijms262211222 (PMC12653303; doi:10.3390/ijms262211222)
Supplement: Supplementary file 1 [file ijms-26-11222-s001.zip › ijms-3958015-supplementary.pdf]

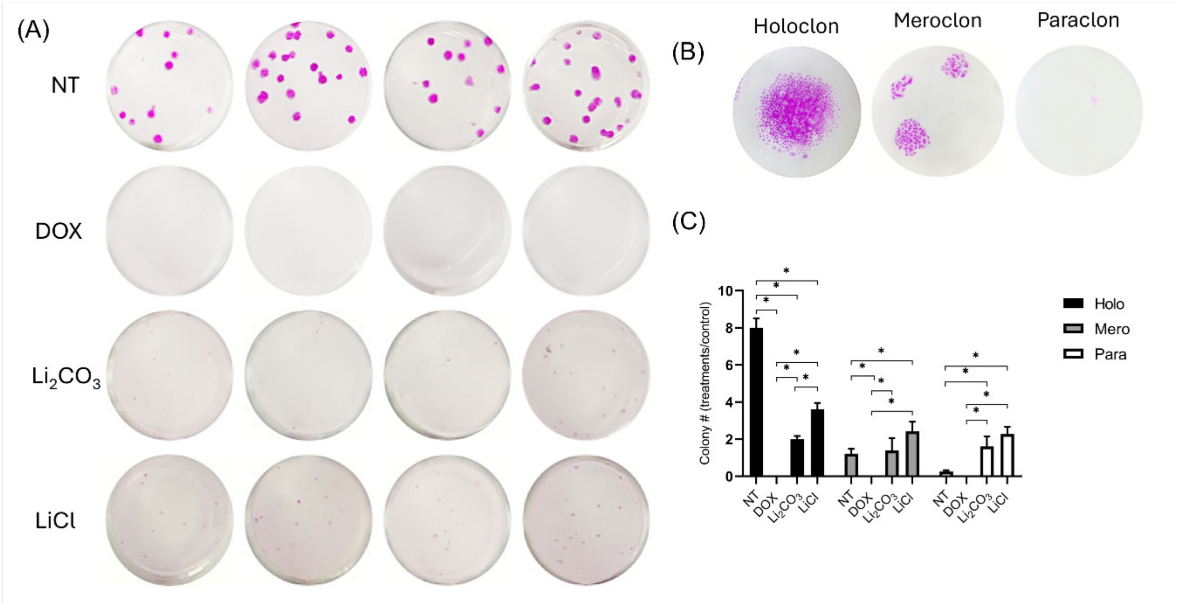

Supplementary Information Figure S1. Clonogenic assay to IC_{50} concentrations of LiCl, Li_2CO_3, and doxorubicin in CRL-1790. (A) Representative photomicrographs of fixed colonies stained with crystal violet, displaying the morphology and density of colorectal cancer cells and clones after 10 days of culture with or without treatment using lithium salts or doxorubicin. (B) Representative images showing the morphological features and density of holoclones, meroclones and paraclones. (C) Histogram illustrating the number of colonies corresponding to holoclones, meroclones, and paraclones in cells treated with lithium salts, doxorubicin, or left untreated for 24 hours. *Statistical significance was set at $p<0.05$ according to one-way ANOVA followed by Tukey's multiple comparisons test.

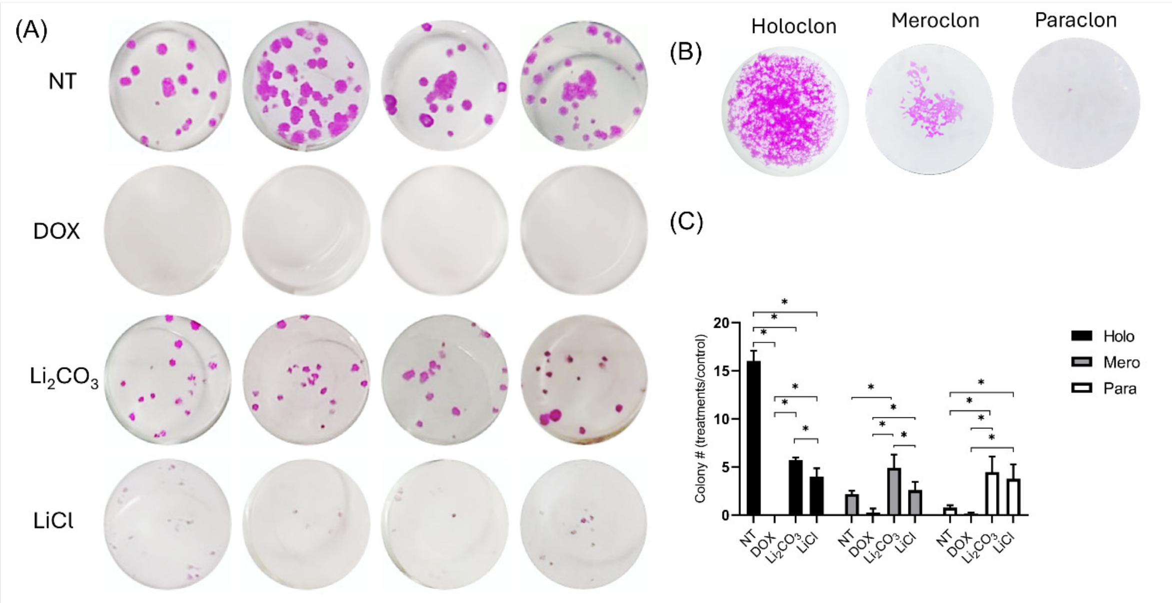

Supplementary Information Figure S2. Clonogenic assay at IC_{50} concentrations of LiCl, Li_2CO_3, and doxorubicin in HCT-116. (A) Representative photomicrographs of fixed colonies stained with crystal violet, displaying the morphology and density of colorectal cancer cells and colonies after 10 days of culture with or without using lithium salts or doxorubicin. (B) Representative images showing the morphological features and density of holoclones, meroclone and paraclones. (C) Histogram illustrating the number of holoclones, meroclones, and paraclones in cells treated with lithium salts, doxorubicin, or left untreated for 24 hours. *Statistical significance was set at $p<0.05$ according to one-way ANOVA followed by Tukey's multiple comparisons test.

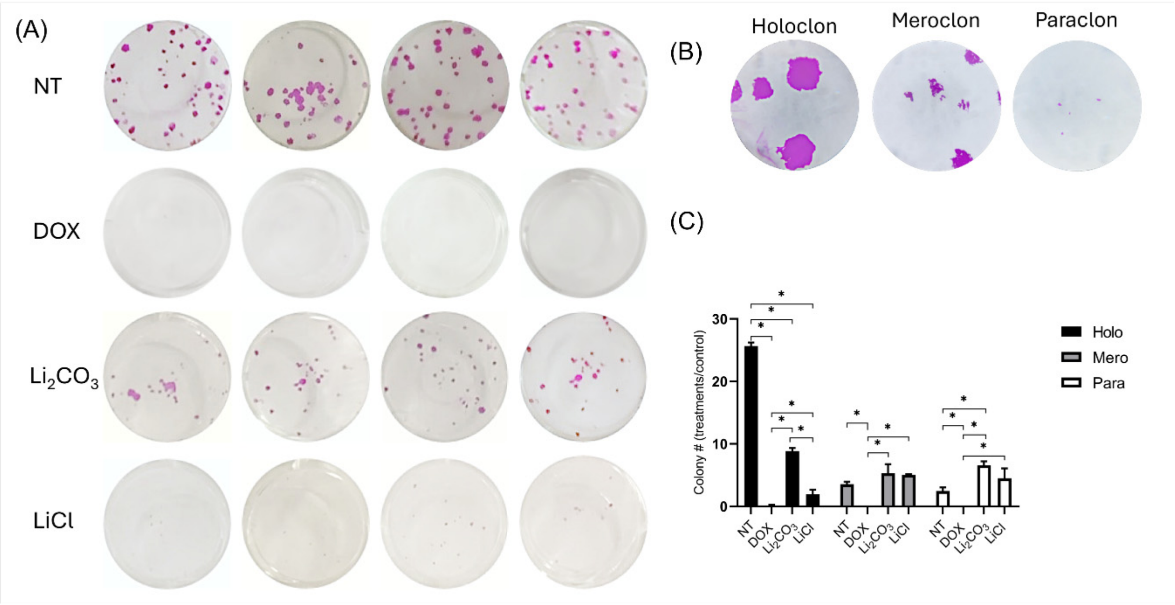

Supplementary Information Figure S3. Clonogenic assay at IC_{50} concentrations of LiCl, Li_2CO_3, and doxorubicin in SW-620. (A) Representative photomicrographs of fixed colonies stained with crystal violet, displaying the morphology and density of colorectal cancer cells and colonies after 10 days of culture under treatment or no treatment with lithium salts or doxorubicin. (B) Representative images showing the morphological features and density of holoclones, meroclones and paraclones. (C) Histogram illustrating the number of colonies for cells holoclones, meroclone and paraclones treated with lithium salts, doxorubicin, and non-treated controls to 24h. *Statistical significance was set at $p<0.05$ according to one-way ANOVA and Tukey's multiple comparisons test.
